# Febrile neutropenia in chemotherapy treated small-cell lung cancer patients

**DOI:** 10.2478/raon-2014-0050

**Published:** 2015-03-25

**Authors:** Renata Rezonja Kukec, Iztok Grabnar, Tomaz Vovk, Ales Mrhar, Viljem Kovac, Tanja Cufer

**Affiliations:** 1 Krka, d.d., Novo mesto, Slovenia; 2 University of Ljubljana, Faculty of Pharmacy, Ljubljana, Slovenia; 3 Institute of Oncology Ljubljana, Ljubljana, Slovenia; 4 University Clinic Golnik, Golnik, Slovenia

**Keywords:** small cell lung cancer, platinum-etoposide chemotherapy, etoposide, febrile neutropenia, plasma drug concentration

## Abstract

**Background.:**

Chemotherapy with platinum agent and etoposide for small-cell lung cancer (SCLC) is supposed to be associated with intermediate risk (10–20%) of febrile neutropenia. Primary prophylaxis with granulocyte colony-stimulating factors (G-CSFs) is not routinely recommended by the treatment guidelines. However, in clinical practice febrile neutropenia is often observed with standard etoposide/platinum regimen. The aim of this analysis was to evaluate the frequency of neutropenia and febrile neutropenia in advanced SCLC patients in the first cycle of standard chemotherapy. Furthermore, we explored the association between severe neutropenia and etoposide peak plasma levels in the same patients.

**Methods.:**

The case series based analysis of 17 patients with advanced SCLC treated with standard platinum/etoposide chemotherapy, already included in the pharmacokinetics study with etoposide, was performed. Grade 3/4 neutropenia and febrile neutropenia, observed after the first cycle are reported. The neutrophil counts were determined on day one of the second cycle unless symptoms potentially related to neutropenia occurred. Adverse events were classified according to Common Toxicity Criteria 4.0. Additionally, association between severe neutropenia and etoposide peak plasma concentrations, which were measured in the scope of pharmacokinetic study, was explored.

**Results.:**

Two out of 17 patients received primary GCS-F prophylaxis. In 15 patient who did not receive primary prophylaxis the rates of both grade 3/4 neutropenia and febrile neutropenia were high (8/15 (53.3%) and 2/15 (13.3%), respectively), already in the first cycle of chemotherapy. One patient died due to febrile neutropenia related pneumonia. Neutropenic events are assumed to be related to increased etoposide plasma concentrations after a standard etoposide and cisplatin dose. While the mean etoposide peak plasma concentration in the first cycle of chemotherapy was 17.6 mg/l, the highest levels of 27.07 and 27.49 mg/l were determined in two patients with febrile neutropenia.

**Conclusions.:**

Our study indicates that there is a need to reduce the risk of neutropenic events in chemotherapy treated advanced SCLC, starting in the first cycle. Mandatory use of primary G-CSF prophylaxis might be considered. Alternatively, use of improved risk models for identification of patients with increased risk for neutropenia and individualization of primary prophylaxis based on not only clinical characteristics but also on etoposide plasma concentration measurement, could be a new, promising options that deserves further evaluation.

## Introduction

Small cell lung cancer (SCLC) accounts for approximately 13% of all lung cancer diagnoses. It is very aggressive, growing rapidly and spreading early. Seventy percent of SCLC patients have extensive disease at the time of diagnosis. The standard therapeutic approach for extensive disease is chemotherapy with platinum agent and topoisomerase II inhibitor etoposide.^1^

Chemotherapy causes haematological as well as non-haematological adverse drug reactions. The most serious haematological toxicity is neutropenia, which can cause fatal septicaemia by suppressing the production of neutrophils and by cytotoxic effects on the cells that line the gastrointestinal tract allowing bacterial multiplication and invasion.^2^ Febrile neutropenia (FN) is a serious adverse event of chemotherapy characterized as an oral temperature > 38.5 °C or two consecutive readings of > 38 °C for 2 h and an absolute neutrophil count (ANC) < 0.5 × 10^9^/l, or expected to fall below 0.5 × 10^9^/l.^3^ It is associated with high morbidity, mortality, costs, and an increase of the risk for chemotherapy dose delays and/or reductions, or even discontinuation of chemotherapy.^4^,^5^

Primary prophylaxis with granulocyte colony-stimulating factors (G-CSFs), *i.e*. use with first cycle of chemotherapy, has been shown to significantly reduce the risk of FN; however, its use in all patients is not considered cost-effective.^2^,^4^ According to recommendations of the European Organisation for Research and Treatment of Cancer (EORTC)^6^, American Society of Clinical Oncology (ASCO)^7^, National Comprehensive Cancer Network (NCCN)^8^, and European Society for Medical Oncology (ESMO) clinical practice guidelines^9^, the prophylactic G-CSF is recommended when the risk of FN is high (≥ 20%). Treatment-related risk factors classify chemotherapy regimens to high (≥ 20%), intermediate (10–20%), or low risk (< 10%) for developing FN.^4^,^6^ When using a chemotherapy regimen associated with an intermediate (10–20%) risk of FN other factors that may increase the overall risk of FN should be considered in making the decision to use prophylactic G-CSF. Guidelines indicate various risk factors, with an older age included in all four guidelines. Additional factors are history of prior FN, poor performance status (PS) and comorbidities^7^,^8^; for further details see Table 1. Recently, genetic factors which are not mentioned in the guidelines have also been associated with the risk of FN.^4^

According to EORTC and NCCN guidelines etoposide/platinum regimen for SCLC is associated with an intermediate risk of FN, while in the ESMO guidelines which provide only the list of regimens with high risk of FN, etoposide/platinum is not listed. ASCO guidelines do not indicate FN risk for any particular chemotherapeutic regimen. Based on the guidelines, primary prophylaxis with G-CSF in SCLC patients treated with etoposide/ platinum regimen is not recommended without a prior identification of a high risk of FN in each individual patient. However, in routine clinical practice FN seems to be frequent in advanced SCLC patients treated with standard etoposide/platinum regimen, who are not entitled to G-CSF prophylaxis.

To get additional information on febrile neutropenia in a first cycle of standard chemotherapy with etoposide/platinum, a post-planned analysis of the frequency of neutropenia and FN in a case series of patients with advanced SCLC, already included in a clinical trial of etoposide pharmacokinetics, was performed. Furthermore, analysis of association of severe neutropenia with previously measured levels of etoposide peak plasma concentration in the same patients during the first cycle of etoposide/cisplatin has been conducted.

## Patients and methods

### Clinical observation

The post-planned analysis of the frequency and grade of neutropenia and FN was conducted in a case series of patients in the first cycle of standard chemotherapy with etoposide/platinum. These patients were already included in a clinical trial of etoposide pharmacokinetics. Furthermore, association between severe neutropenia and etoposide peak plasma levels was explored.

Eligible patients were at least 18 years old receiving first-line chemotherapy with etoposide/platinum for advanced SCLC confirmed by cytology or histology. Other entry criteria included World Health Organization PS 0–2, adequate haematological parameters and medical conditions allowing chemotherapy, satisfactory liver and renal function. The main exclusion criteria were Gilbert syndrome, Criegler-Najjar syndrome, active gastrointestinal disorders, and concomitant drugs entering the clinically important pharmacokinetic interactions. The Charlson comorbidity index was not assessed; however, patients with some comorbidities, such as liver or kidney dysfunction were excluded by the criteria of pharmacokinetic study. Patients gave written informed consent to participate in the pharmacokinetic study, which was approved by the Slovenian Ethics Committee for Research in Medicine (approval ref. no. 02/11/11) and was carried out according to the Helsinki Declaration.

Patients received a standard myelosuppressive chemotherapeutic regimen of etoposide and cisplatin or carboplatin without any concurrent irradiation. G-CSF prophylaxis was administered according to current guidelines. Planned dose of etoposide was of 100 mg/m^2^ intravenously on day 1 through 3. Cisplatin or carboplatin were administered intravenously on day 2 at a planned dose of 80 mg/m^2^ or at a target area under the curve (AUC) 5–6 mg min/ml (maximally 350 mg/m^2^), respectively. Patients were followed according to routine practice guidelines valid at that period at our university clinic. Neutrophil count was determined on day one of the next 3-week cycle, or earlier in case of clinical symptoms associated with neutropenia. If indicated, patients with severe neutropenia and/ or FN were hospitalized at our clinic. Grade 3/4 neutropenia and FN were classified according to Common Toxicity Criteria (NCI-CTC, version 4.0).

The reason for including only the first chemo-therapy cycle in our post-planned analysis was relatively high rate of observed neutropenia grade 3/4 or FN in the first cycle while using primary G-CSF prophylaxis according to current guidelines. The following cycles were not included into our analysis due to the fact that G-CSF prophylaxis had been used in the majority of patients in consecutive cycles. In addition, some patients in consecutive cycles received decreased etoposide dose or administration of chemotherapy was delayed.

### Pharmacokinetic sampling and drug assay in the scope of pharmacokinetic study

Blood sampling was performed on days 1, 2 and 3 in the first cycle of chemotherapy. Blood samples (6 ml) were collected at the end of etoposide 60-min infusion. Samples were immediately placed on ice. Plasma was separated by centrifugation at 3000 × g and 4 °C for 10 min and stored at −80 °C until the analysis. Etoposide plasma concentration was determined by high-performance liquid chromatography with fluorimetric detection using a modified method of Krogh-Madsen *et al*.^10^ Linearity of the method was 0.125–30 mg/l with a lower limit of quantification of 0.125 mg/l. The method was accurate (all deviations ≤ 10.3%) and reproducible (coefficient of variability ≤ 7.22% intra-day and ≤ 7.33% inter-day).

## Results

According to patients baseline characteristics presented in Table 2 our group of 17 patients represents a typical population of advanced SCLC patients treated with chemotherapy, with a mean age of 64.1 years (range, 51–78 years), mostly males (76.5%) and PS ≤ 2. In the first cycle etoposide was administered in a full dose in 13 of all patients (76.5%). Primary G-CSF prophylaxis was administered in only 2 patients (11.8%).

Two out of 17 cases (11.8%) of FN have been observed in the first cycle, one of these two patients died due to FN related pneumonia. Taken into account only 15 patients without primary prophylaxis with G-CSF the rate of FN was even higher, *i.e*. 13.3% (2/15). The whole rate of neutropenia grade 3/4 after the first cycle was also quite high, it was recorded in 8 out of 15 patients not receiving primary G-CSF prophylaxis (53.3%). Of note, in our study neutrophil count has only been determined on day one of the second cycle, unless symptoms potentially related to neutropenia occurred.

In addition, mild grade 1/2 neutropenia or normal neutrophil blood count have been observed in 7/15 (46.7%) of patients without G-CSF prophylaxis on the scheduled day of the second cycle.

Mean etoposide peak plasma concentration in the first cycle of chemotherapy was 17.6 mg/l (from 10.59 to 27.49 mg/l) (Table 2). Of note, the highest levels 27.07 and 27.49 mg/l were determined in two patients with FN. Patients with grade 3/4 neutropenia not experiencing FN had also high mean peak plasma concentrations of 17.4 mg/l (from 14.43 to 23.71 mg/l). Mean etoposide peak plasma concentration in patients with grade 1/2 neutropenia was 16.84 (from 16.17 to 17.88 mg/l), while patients who did not experience neutropenia had etoposide plasma level of 14.5 mg/l (from 10.59 to 20.25 mg/l).

## Discussion

According to the guidelines, G-CSF primary prophylaxis is mandatory when the overall risk of FN due to chemotherapy regimen and other factors is ≥ 20%. Etoposide/platinum regimen for SCLC treatment is considered to be associated with 10–20% risk of FN and G-CSF primary prophylaxis is not unambiguously recommended by current guidelines.^6^–^9^ We reviewed studies on the basis of which guidelines classified etoposide/platinum regimen for SCLC treatment into the intermediate risk group for FN.

Taken together, according to EORTC, ASCO, NCCN and ESMO guidelines, information on FN rates in SCLC patients treated with etoposide/ platinum regimen is scarce. Only three published studies related to the risk of FN in SCLC patients treated by etoposide/cisplatin are cited^11^–^13^, two of them^12^,^13^ are even very likely the same study. Roth *et al*.^11^ reported grade 3/4 granulocytopenia in 70% of patients, while in other two studies^12^,^13^ grade 3/4 neutropenia was not even reported. FN was not reported in any of these studies.^11^–^13^ Of note, in all of these trials concomitant irradiation has been performed in selected patients (Table 3).

Therefore, we performed a comprehensive PubMed literature search to find additional data on grade 3/4 neutropenia and FN rates in SCLC patients treated with first-line intravenous etoposide/ platinum regimen (etoposide dosage 240 to 420 mg/m^2^ per cycle) without concurrent radiotherapy and G-CSF primary prophylaxis. In addition to the above 3 mentioned articles^11^–^13^, our literature search found nine studies^14^–^22^ (Table 4). In fact, our search confirmed a substantially high rate of grade 3/4 neutropenia (51–91%) observed in SCLC patients treated with etoposide/platinum chemotherapy given the fact that G-CSF use has been allowed in 3 out of nine trials. In addition, FN rates reported in five of these nine articles^14^–^22^ were in the range of 10–20% referred in the guidelines.^6^,^8^ The reported rates of FN during all, not only the first cycle of the chemotherapy, were in the range from 9.5% to 17%, with the highest rate observed in the trial using relatively high daily dose of etoposide, *i.e.* 140 mg/m^2^ for 3 days.^16^,^18^,^19^,^21^,^22^ Of note, data on neutropenia rates were based on all chemotherapy cycles and not just the first cycle.

In our limited series of patients, severe neutropenia G3/4 and FN were observed in unexpectedly high portion of patients not receiving primary G-CSF prophylaxis already in the first cycle of platinum/etoposide chemotherapy; neutropenia G 3/4 developed in more than half patients (53.3%) and FN developed in 2 out of 15 patients. Of note, neutropenia and FN were recorded after the first cycle of the chemotherapy based on the neutro-phil count determined only on day one of the second cycle, unless symptoms potentially related to neutropenia occurred. In addition, only 12 out of these 15 patients without G-CSF prophylaxis received the full dose of etoposide. Patient 4 was on long-term treatment with corticosteroids. This patient developed FN with lung infection and died. Taken together, more than half of our patients not receiving primary G-CSF prophylaxis developed at least grade 3/4 neutropenia already in the first cycle, with FN representing a quarter of these eight patients. None of the patients on primary G-CSF prophylaxis developed grade 3/4 neutropenia. Based on this observation most of our consecutive patients included into the prospective etoposide pharmacokinetic study received primary GCS-F prophylaxis and are not included in this analysis.

Compared to the literature search data showing the rate of grade 3/4 neutropenia between 51 and 91% and FN rate between 9.5 and 17% after all cycles in the population of patients not receiving primary prophylaxis with G-CSF the 53.3% rate of grade 3/4 neutropenia and 13.3% rate of FN observed in our patients already in the first cycle without G-CSF prophylaxis is rather high. Taking into account 4 additional patients with grade 1/2 neutropenia recorded on the day one of the second cycle (including one patient taking corticosteroids chronically), the number of grade 3/4 neutropenia in the first cycle might be even higher, if the ANC was measured in the middle of the first chemotherapy cycle.

Despite the fact that the majority of our patients did not classify to high risk FN due to first-line chemotherapy, no concurrent palliative irradiation, good PS, no major comorbidities and normal kidney, liver and bone marrow function, which were all prerequisites for patients to be included into the pharmacokinetic trial, the rate of FN and 3/4 neutropenia observed after first cycle of the chemotherapy was substantially high. The reason for this might be in the fact that half of our patients were older than 65 years and all of them had advanced disease. Age more than 65 years has not been taken as high-risk criteria per se in our selected population of patients without comorbidities and with a good PS included into the pharmacokinetic trial. Obviously in elderly, fragile population the use of comprehensive geriatric assessment might improve our efforts to better identify patients with an increased risk of cytotoxic drugs complications.^23^ However, so far there are no reported prognostic validation studies using comprehensive geriatric assessment for decision on prophylactic use of G-CSF. In addition, we have still not found a score that would help us select these patients in a more comprehensive fashion.

EORTC, ASCO, NCCN and ESMO guidelines indicate various risk factors that predispose to increased risk of FN. Older age is the only factor included in all four guidelines. EORTC guidelines define older age even as patient-related risk factor most consistently associated with an increased FN risk.^6^ However, Crawford *et al*. tested various patient’s baseline characteristics as possible risk factors for ≥ 1 event of FN, including age, body weight, body surface area, sex, PS, disease stage, and neoplastic disease involvement in the marrow. Surprisingly, only sex was marginally predictive in their study, while patient age was not found to be a risk factor for FN.^24^

The association between neutropenic events and etoposide peak plasma concentration has been well perceived by our analysis. According to the literature etoposide therapeutic trough serum concentration range in cancer patients is 2 to 6 mg/l and peak, 8 to 14 mg/l.^25^ In all our groups of patients, *i.e*. patients with FN, grade 3/4 neutropenia, grade 1/2 neutropenia and without neutropenia, mean peak plasma concentration of etoposide was above therapeutic level (*i.e*. 14 mg/l). However, relatedness of mean peak plasma concentration height with severity of neutropenia was observed; concentrations were the highest in patients with FN and declined to the lowest levels observed in patients without neutropenia. Based on the fact that the mean etopo-side peak plasma concentration was above therapeutic level also in patients without neutropenia could be anticipated that the frequency of (high-grade) neutropenia would be even higher if neutrophils were measured at the time of the largest expected neutrophil nadir.

On another point, peak plasma etoposide concentrations in two patients (one of them did not receive G-CSF prophylaxis) not experiencing neutropenia were within therapeutic range. Interestingly enough, in patient 7 etoposide plasma concentration was increased (14.73 mg/l) after dosage of etoposide; however, primary G-CSF prophylaxis was received and neutropenia did not develop. These data raised the question of whether high plasma concentrations measured immediately after the first application of etoposide on day one of the three day application course could help in selection of patients for primary G-CSF prophylaxis.

Our analysis is limited by the biases of selected patient population with good PS, without major comorbidities, treated in a controlled situation in the frame of the prospective clinical study. Additionally, the number of the patients is low and neutrophil counts were routinely measured only on the day one of the second cycle and not at the time of the largest expected neutrophil nadir in the middle of the cycle. But, all these limitations do not compromise our conclusion that the risk of FN in advanced SCLC population of patients treated with etoposide/platinum is substantially high. In a real world scenario the probability of FN in these patients might be even higher.

The goal is to develop a comprehensive risk models for FN which can be used as a guide whether or not to incorporate primary G-CSF prophylaxis for each individual patient.^26^,^27^ Some predictive models for neutropenia in the first cycle have already been proposed. However, a prospective study is needed for their validation. On the other hand, individualization of etoposide dosage taking into account pharmacokinetic parameters as well as genetic factors such as genetic polymorphisms, which can also affect drug plasma concentrations, is another option that has to be considered.^28^

## Conclusions

According to the guidelines etoposide/platinum regimen for SCLC treatment is not associated with high ≥ 20% risk of FN and primary G-CSF prophylaxis is therefore not mandatory. However, in our case series analysis of selected advanced SCLC patients included in a prospective pharmacokinetic trial, the rate of neutropenic complications in patients not receiving primary G-CSF prophylaxis was substantially high, already in the first cycle. Advanced SCLC patients treated with a standard dose of etoposide in combination with platinum may have increased plasma etoposide concentrations as reported in our patients and may therefore be at increased risk for high grade neutropenia and FN.

There is a need of greater effort to reduce the risk of neutropenic events starting in the first cycle. To avoid overuse of G-CSF a better prediction of post-chemotherapy neutropenic events, based on etoposide peak plasma concentration, might be of great value. An option could be the development and validation of risk models for severe neutropenia, based on etoposide plasma concentration on day one of the first cycle, a strategy that deserves further evaluation.

## Figures and Tables

**TABLE 1.** Factors associated with FN risk according to EORTC, ASCO, NCCN and ESMO guidelines

**Risk factor**	**EORTC**	**ASCO**	**NCCN**	**ESMO**
Older age (≥ 65 years)	■	■	■	■
Comorbidities	Liver, renal or cardiovascular diseases	■	Liver dysfunction, poor renal function	
History of prior FN	■	■	■	
Poor performance status	■	■	■	
Extensive prior treatment including large radiation ports		■	■	Reduced marrow reserve (e.g. ANC < 1.5 × 10^9^/l) due to radiotherapy of > 20% marrow
Poor nutritional status	■	■		
Advanced stage of disease	■	■		
Cytopenias due to bone marrow involvement by tumour		■	■	
The presence of open wounds or active infections		■	■	
Lack of antibiotic prophylaxis	■			
Lack of G-CSF use	■			
Female gender	■			
Haemoglobin < 12 g/dl	■			
Administration of combined chemoradiotherapy		■		
Previous chemotherapy			■	
Pre-existing neutropenia			■	
Recent surgery			■	
Further infections in the next treatment cycle considered life-threatening				■
Dose reduction below treshold				■
Delay of chemotherapy				■
Lack of protocol adherence if compromising cure rate, overall or disease-free survival				■
Human immunodeficiency virus				■

ANC = absolute neutrophil count; ASCO = American Society of Clinical Oncology; EORTC = European Organisation for Research and Treatment of Cancer; ESMO = European Society for Medical oncology; FN = febrile neutropenia; G-CSF = granulocyte colony-stimulating factor; NCCN = National Comprehensive Cancer Network

**TABLE 2.** Patients and treatment characteristics with the data on grade 3/4 and febrile neutropenia in the first cycle

**Patient n = 17**	**Age** **Mean (range)**	**Sex**	**PS**	**Etoposide dose (%)**	**Neutropenia grade**	**FN** **Yes/No**	**G-CSF prophylaxis**	**Etoposide peak plasma concentration (3 days mean) (mg/l)**
1	60	F	1	100	2	No	No	16.27
2	62	M	0	100	4	No	No	14.43
3	65	M	1	100	1	No	No	16.17
4	60	M	1	100	4	Yes, death	No	27.07
5	64	F	0	100	4	Yes	No	27.49
6	78	M	1	75	0	No	No	15.09
7	51	M	1	100	0	No	Yes	14.73
8	73	M	1	100	1	No	No	17.04
9	63	M	1	100	1	No	No	17.88
10	78	M	1	75	0	No	No	11.93
11	62	M	1	75	0	No	Yes	10.59
12	54	M	0	75	3	No	No	15.14
13	63	F	1	100	3	No	No	17.65
14	64	M	1	100	4	No	No	16.73
15	65	F	0	100	0	No	No	20.25
16	66	M	0	100	4	No	No	23.71
17	62	M	1	100	3	No	No	16.74

	**64.1 (51–78)**				**Grade 3/4: 8/17 (47.1%)** **Grade 3/4 (no G-CSF): 8/15 (53.3%)** **Grade 1/2 or 0:9/17 (52.9%)** **Grade 1/2 or 0 (no G-CSF): 7/15 (46.7%)**	**2/17 (11.8%)** **No G-CSF 2/15 (13.3%)**		**17.6 (range 10.59–27.49)**

FN = febrile neutropenia; G-CSF = granulocyte colony-stimulating factor; PS = performance status; M = male; F = female

**TABLE 3.** A summary of studies reporting risk of FN by ASCO, EORTC and NCCN guidelines

**Reference, year**	**No of patients entered**	**Treatment regimen**	**% pts with grade 3/4 neutropenia**	**% pts with FN**	**Concurrent radiotherapy**	**G-CSF**
Roth *et al*.^11^, 1992	159	Etoposide 80 mg/m^2^/d i.v. for 5 days, Cisplatin 20 mg/m^2^/d i.v. for 5 days, every 3 weeks, 4 cycles	70 (granulocytopenia)	NR	Yes (patients with brain metastases).	No.
Skarlos *et al*.^12^, 1994	Regimen A: 73	Regimen A: Etoposide 100 mg/m^2^/d i.v. days 1-3, Cisplatin 50 mg/m^2^/d day 1 to 2	NR	NR	Yes (responding limited disease patients and complete responders with extensive disease)	No.
	Regimen B: 74	Regimen B: Etoposide 100 mg/m^2^/d i.v. days 1–3, Carboplatin 300 mg/m^2^/d i.v. day 1, every 3 weeks, 6 cycles				
Kosmidis *et al*.^13^, 1994	Regimen A: 73	Regimen A: Etoposide 100 mg/m^2^/d i.v. days 1–3, Cisplatin 50 mg/m^2^/d day 1 to 2	NR	NR	Yes (limited disease patients)	No.
	Regimen B: 74	Regimen B: Etoposide 100 mg/m^2^/d i.v. days 1–3, Carboplatin 300 mg/m^2^/d i.v. day 1, every 3 weeks, 6 cycles				

ASCO = American Society of Clinical Oncology; EORTC = European Organisation for Research and Treatment of Cancer; FN = febrile neutropenia; NCCN=National Comprehensive Cancer Network; G-CSF = granulocyte colony-stimulating factor; i.v. = intravenous administration; NR = not reported; pts = patients

**TABLE 4.** A summary of comprehensive literature search of studies on FN and grade 3/4 neutropenia

**Reference, year**	**No of patients eligible for evaluation**	**Treatment regimen**	**G3/4 neutropenia(% of pts)**	**FN (% of pts)**	**G-CSF prophylaxis**
Miller *et al*.^14^, 1995	156	Etoposide 130 mg/m^2^/d i.v. for 3 days, cisplatin 25 mg/m^2^/d i.v. for 3 days, every 3 weeks, up to 8 cycles	85.0	NR	No
Pujol *et al*.^15^, 2001	109	Etoposide 100 mg/m^2^ i.v. days 1–3, cisplatin 100 mg/m^2^ i.v. day 1, every 4 weeks, up to 6 cycles	91.0	NR	No
Quoix *et al*.^16^, 2001	38	Etoposide 100 mg/m^2^ i.v. days 1–3, carboplatin AUC 5 mg/ml/min day 1, every 4 weeks, up to 6 cycles	57.0% cycles(NR per patient)	13.2	No
Schiller *et a*l.^17^, 2001	402	Etoposide 120 mg/m^2^ i.v. days 1–3, cisplatin 60 mg/m^2^ i.v. day 1, every 3 weeks, 4 cycles	67.0	NR	Used at the discretion of the treating physician. (no data on use)
Hanna *et al*.^18^, 2006	106	Etoposide 120 mg/m^2^ i.v. days 1–3, cisplatin 60 mg/m^2^ i.v. day 1, every 3 weeks, at least 4 cycles	86.5	10.4	Used in accordance with their package inserts or the 1999 guidelines from the ASCO. (no data on use)
Schmittel *et al*.^19^, 2006	35	Etoposide 140 mg/m^2^ i.v. days 1–3, carboplatin AUC 5 mg min/ml day 1, up to 6 cycles	51.0	17.0	No
Heigener *et al*.^20^, 2009	37	Etoposide 140 mg/m^2^ i.v. days 1–3, carboplatin AUC 5 i.v. day 1, every 4, up to 6 cycles	69.4	NR	No
Lara *et al*.^21^, 2009	324	Etoposide 100 mg/m^2^ i.v. days 1–3, cisplatin 80 mg/m^2^ i.v. day 1, every 3 weeks, 4 cycles	68.0	9.5	Use of G-CSF was allowed per investigator discretion. (no data on use)
Zatloukal *et al*.^22^, 2010	203	Etoposide 100 mg/m^2^ i.v. days 1–3, cisplatin 80 mg/m^2^ i.v. day 1, every 3 weeks, 6 cycles	59.6	9.9	No
			**Grade 3/4 (range): 51.0–91.0**	**FN (range): 9.5–17.0**	

ASCO=American Society of Clinical Oncology; d = day; FN = febrile neutropenia; G-CSF = granulocyte colony-stimulating factor; i.v. = intravenous administration; NR = not reported; pts = patients;
